# Managing microbiomes for human health: An annotated selection of World Wide Web sites relevant to the topics in *microbial biotechnology*

**DOI:** 10.1111/1751-7915.13271

**Published:** 2018-04-16

**Authors:** Lawrence P. Wackett

**Affiliations:** ^1^ Department of Biochemistry, Molecular Biology & Biophysics BioTechnology Institute University of Minnesota St. Paul MN 55108 USA

## The microbiome

http://themicrobiome.com/en/

The microbiome portal is a general clearinghouse of information pertaining to human microbiome research.

## Managing human gut microbiomes: Podcast

https://soundcloud.com/sydney-ideas/gut-microbiome-a-new-target-for-managing-human-metabolic-health

This site hosts a podcast for a talk on managing gut microbiomes to promote human health.

## Human microbiome: Publications using Illumina sequencing

https://www.illumina.com/documents/products/research_reviews/metagenomics_research_review.pdf

This link is to a commercial brochure put out by the company Illumina, a major player in DNA sequencing equipment. The brochure is well‐prepared and highlights important publications dealing with analyzing microbiomes for their impact on human health.

## Gut microbiome as a clinical tool

https://www.nature.com/articles/nrgastro.2017.29

This review article poses the question as to if and when it will be possible to modulate gut microbiomes to better manage gastrointestinal diseases.

## Microbiomes and human health: Special issue

http://www.mdpi.com/journal/nutrients/special_issues/microbiome-human-health

This webpage is the entry point to a special journal issue covering different aspects of microbiomes and human health.

## NIH human microbiome project

https://hmpdacc.org

This major page of the U.S. National Institutes of Health deals with their funded human microbiome projects. The data consists of analyzing five different major body sites and using 16S and shotgun sequencing methods.

## NIH commonfund microbiome site

https://commonfund.nih.gov/hmp

This site from the U.S. National Institutes of Health hosts research resources to aid in the study of human microbiomes and their role in health and disease.

## EU MetaHIT

http://www.metahit.eu

MetaHIT stands for metagenomics of the human intestinal tract and this is the main website for this project funded by the European Commission with 13 partners from industry and universities.

## Human microbiome project: Broad Institute

https://www.broadinstitute.org/hmp/human-microbiome-project

This site describes the human microbiome projects of the Broad Institute of MIT and Harvard University.

## Human microbiome and forensics

https://onlinelibrary.wiley.com/doi/full/10.1111/1751-7915.12699

This review article highlights the potential for using human microbiome data in forensics and criminal investigations.

## Standards for human fecal sampling processing

https://www.nature.com/articles/nbt.3960

In all metagenomic studies, sampling methods are critical to obtain representative DNA for sequencing. Differences in sampling can yield different results and so this study addresses standardization of sampling methods.

## Microbiome: Biotech primer

https://weekly.biotechprimer.com/the-microbiome-magnified/

This site lists and describes some of the commercial players in developing human microbiome science.

## Microbiomes; Twelve companies to watch

http://blog.signals-analytics.com/microbiome-on-the-rise-12-companies-to-watch-1

This page tracks a good number of companies involved in human microbiome science and shows where their products are in the developmental cycle.

## Enterome

http://www.enterome.fr

Enterome is a company focused on the development of microbiome‐derived biomarkers and drugs for treating cancer.

## Microbiomes: Biotech

https://www.fiercebiotech.com/topic/microbiome

This is a general site following business developments at many companies focused on human microbiome research and development.

## Top microbiome companies

https://www.ventureradar.com/keyword/Microbiome

This site covers human microbiome companies from the perspective of the venture funding community.

## Microbiome therapeutics

http://www.mbiome.com

This is the site for a company that is focused on therapies to maintain health by modulating the human gastrointestinal microbiome.

## Evolve BioSystems

https://www.evolvebiosystems.com

This company is focused on therapies to positively modulate the newborn gut microbiome, that plays a critical role in proper immune and metabolic development.

## Vedanta Biosciences

https://www.vedantabio.com

Vedanta Biosciences is involved in the rational design of medicines based on human commensal bacteria.

## Seres Therapeutics

http://www.serestherapeutics.com

Sera Therapeutics is looking to use live bacteria to treat human diseases.

